# Supplementary figures and images for: Surface Topography, Microbial Adhesion, and Immune Responses in Silicone Mammary Implant-Associated Capsular Fibrosis
Source: Int J Mol Sci. 2024 Mar 9;25(6):3163. doi: 10.3390/ijms25063163 (PMC10969816; doi:10.3390/ijms25063163)

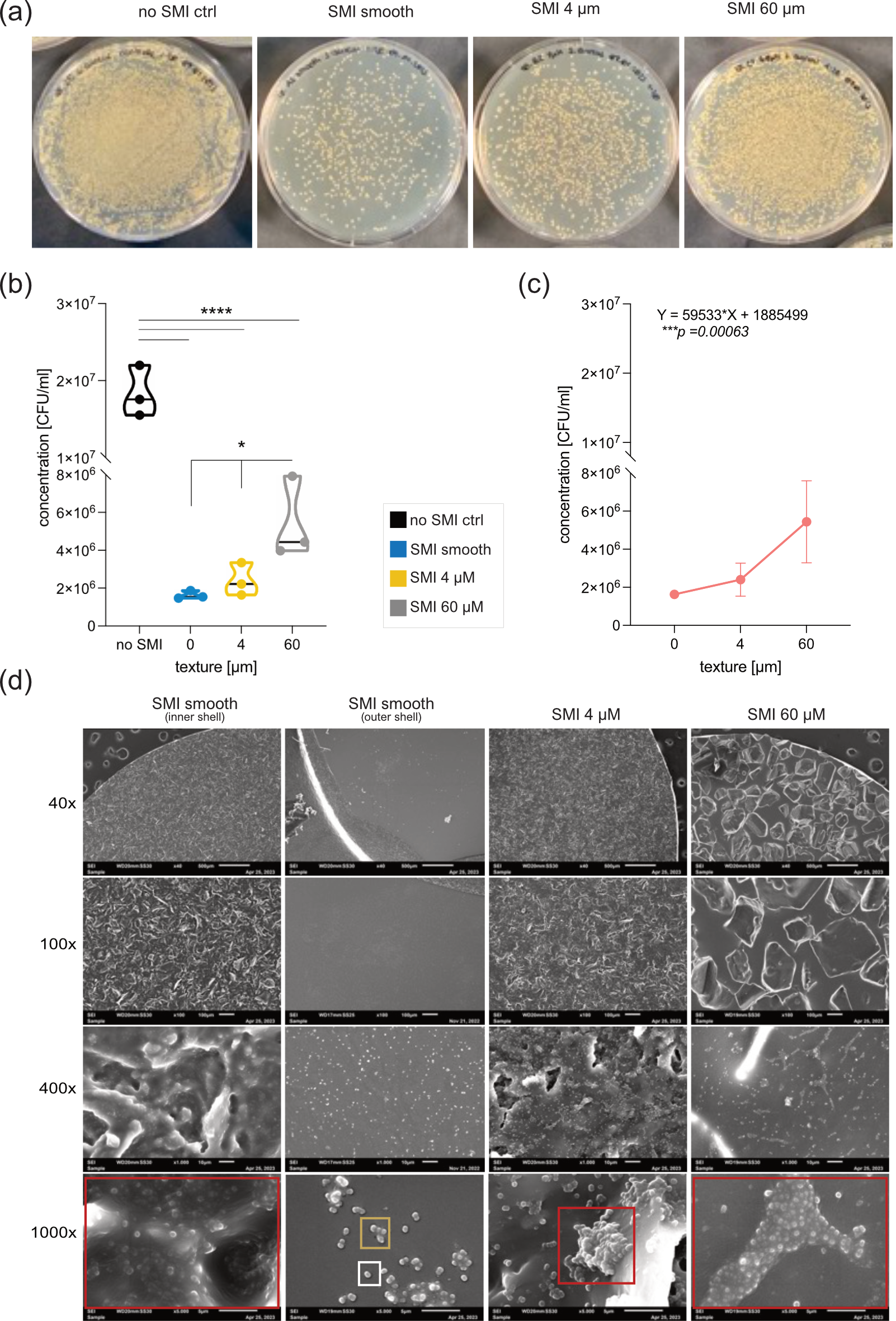

Supplement: Supplementary file 1 [file ijms-25-03163-s001.zip › Figure S1_SA.png]

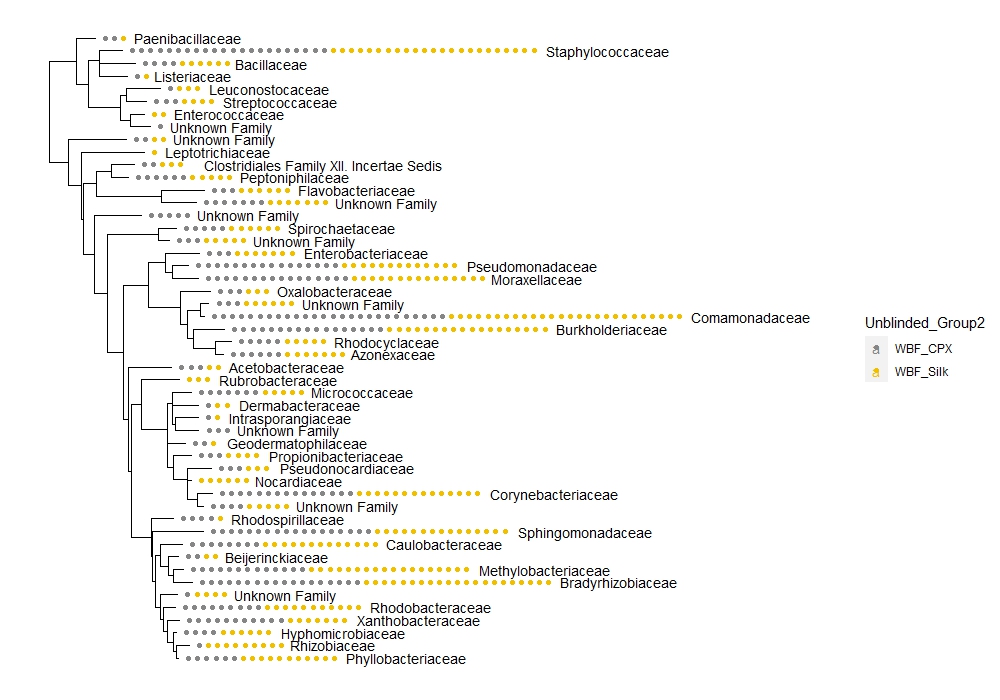

Supplement: Supplementary file 1 [file ijms-25-03163-s001.zip › Figure S2_WBF.jpeg]

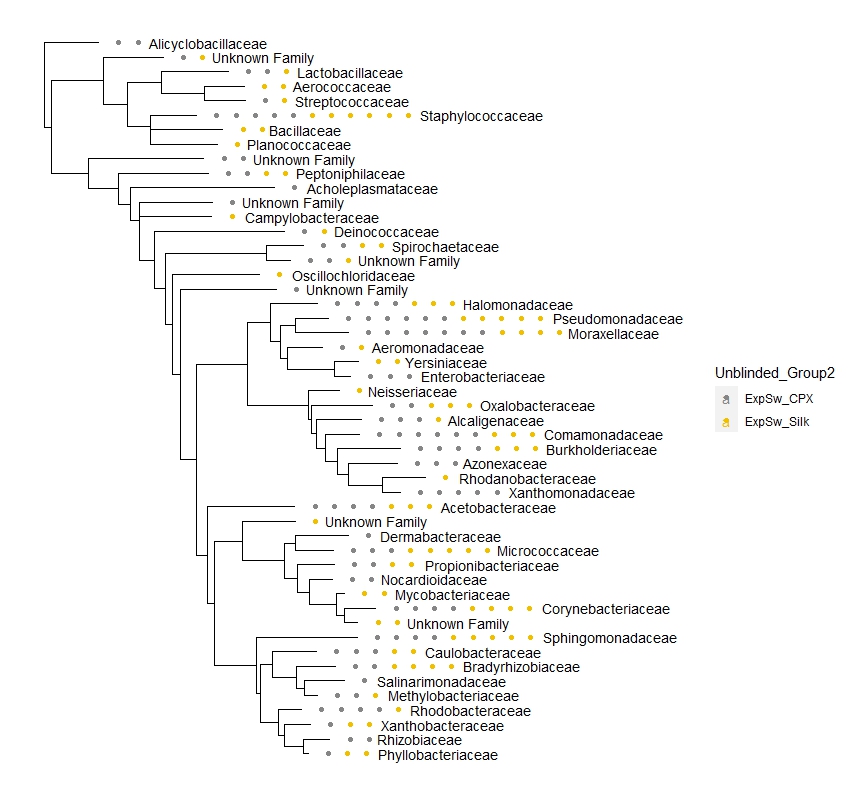

Supplement: Supplementary file 1 [file ijms-25-03163-s001.zip › Figure S3_ExpSurf.jpeg]

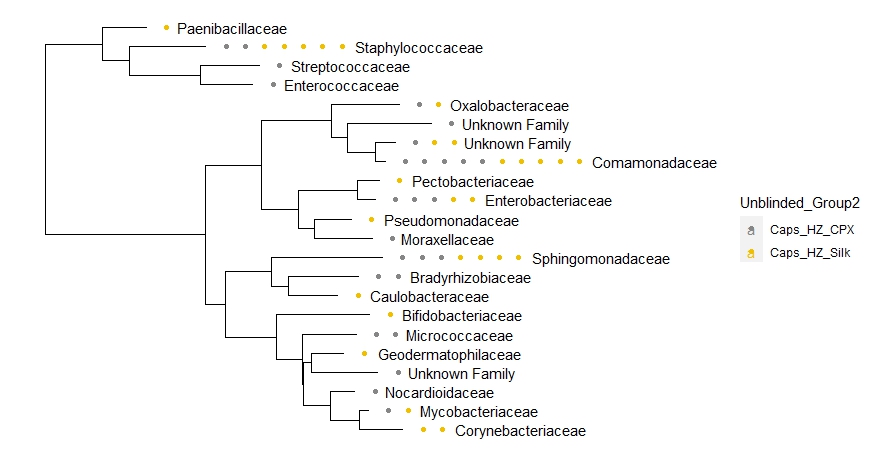

Supplement: Supplementary file 1 [file ijms-25-03163-s001.zip › Figure S4_CT.jpeg]

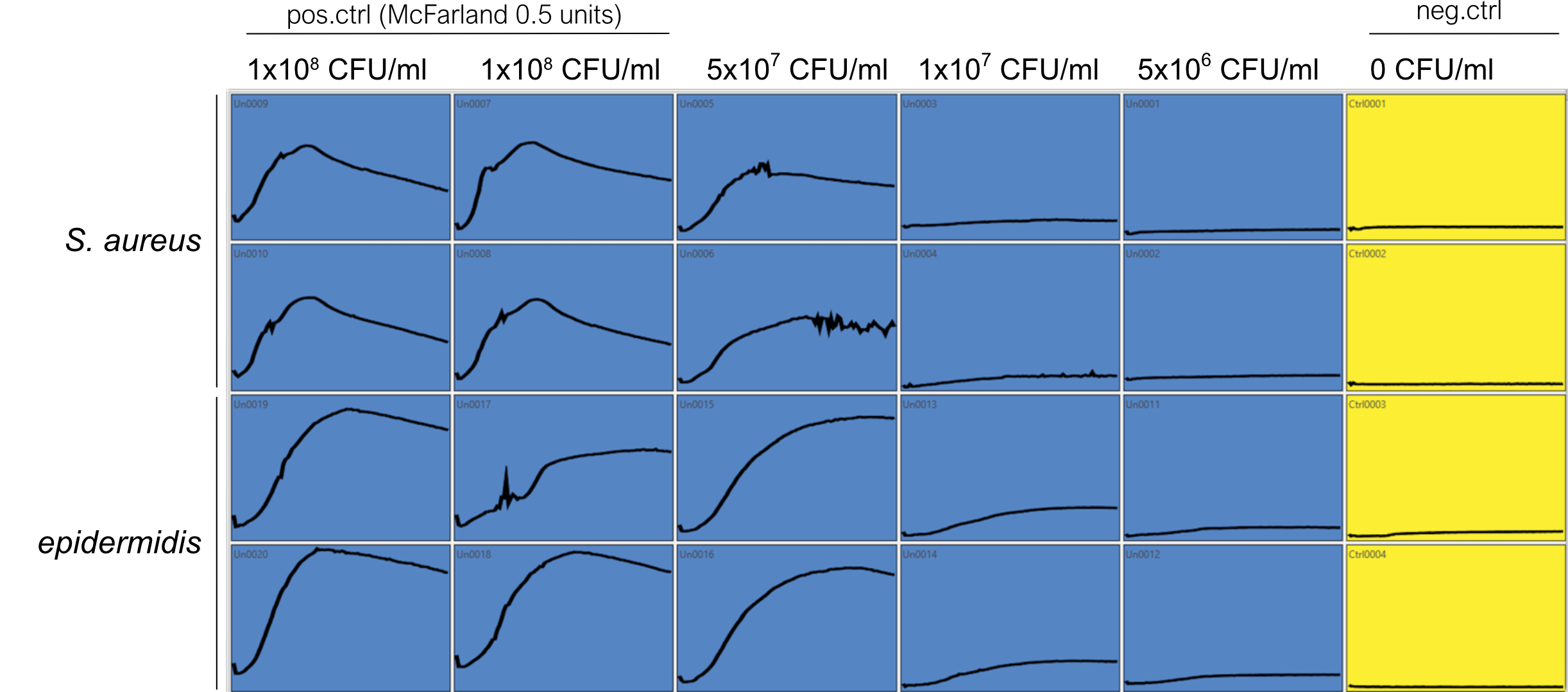

Supplement: Supplementary file 1 [file ijms-25-03163-s001.zip › Supplementary Figure S5.jpg]
